# Targeting Iron Acquisition Blocks Infection with the Fungal Pathogens *Aspergillus fumigatus* and *Fusarium oxysporum*

**DOI:** 10.1371/journal.ppat.1003436

**Published:** 2013-07-11

**Authors:** Sixto M. Leal, Sanhita Roy, Chairut Vareechon, Steven deJesus Carrion, Heather Clark, Manuel S. Lopez-Berges, Antonio diPietro, Marcus Schrettl, Nicola Beckmann, Bernhard Redl, Hubertus Haas, Eric Pearlman

**Affiliations:** 1 Department of Ophthalmology and Visual Sciences, Case Western Reserve University, Cleveland, Ohio, United States of America; 2 Department of Pathology, Case Western Reserve University, Cleveland, Ohio, United States of America; 3 Department of Genetics, Universidad de Cordoba, Cordoba, Spain; 4 Department of Molecular Biology, Medical University of Innsbruck, Innsbruck, Austria; Albert Einstein College of Medicine, United States of America

## Abstract

Filamentous fungi are an important cause of pulmonary and systemic morbidity and mortality, and also cause corneal blindness and visual impairment worldwide. Utilizing *in vitro* neutrophil killing assays and a model of fungal infection of the cornea, we demonstrated that Dectin-1 dependent IL-6 production regulates expression of iron chelators, heme and siderophore binding proteins and hepcidin in infected mice. In addition, we show that human neutrophils synthesize lipocalin-1, which sequesters fungal siderophores, and that topical lipocalin-1 or lactoferrin restricts fungal growth in vivo. Conversely, we show that exogenous iron or the xenosiderophore deferroxamine enhances fungal growth in infected mice. By examining mutant *Aspergillus* and *Fusarium* strains, we found that fungal transcriptional responses to low iron levels and extracellular siderophores are essential for fungal growth during infection. Further, we showed that targeting fungal iron acquisition or siderophore biosynthesis by topical application of iron chelators or statins reduces fungal growth in the cornea by 60% and that dual therapy with the iron chelator deferiprone and statins further restricts fungal growth by 75%. Together, these studies identify specific host iron-chelating and fungal iron-acquisition mediators that regulate fungal growth, and demonstrate that therapeutic inhibition of fungal iron acquisition can be utilized to treat topical fungal infections.

## Author Summary

Fungal pathogens, in addition to causing life-threatening systemic disease, can also invade the cornea and cause blindness and visual impairment. In the current study, we examined the role of iron acquisition in corneal infections caused by *Aspergillus* and *Fusarium*. We first demonstrated that expression of iron chelators, heme, siderophore binding proteins and hepcidin is elevated in infected corneas. Secondly, we showed impaired in vivo growth in *Aspergillus* and *Fusarium* with mutations in the pathway leading to production of iron-binding siderophores. These mutants were also more susceptible to killing by human neutrophils. Based on these observations, we targeted these pathways using topical iron chelators and found that they blocked fungal growth in the cornea. Finally, as statins target the enzyme HMG-CoA reductase, which is required for siderophore and ergosterol biosysnthesis, we found that topical statins inhibited fungal growth and reduced infection, and showed that combined treatment with the iron chelator deferiprone and statins had an additive effect on fungal infection. Together, these studies demonstrate that therapeutic inhibition of fungal iron acquisition can be utilized to treat topical fungal infections.

## Introduction

*Aspergillus* and *Fusarium* are filamentous fungi that cause lethal infections in immune suppressed individuals [1], [2]. Additionally, they infect the corneas of immunocompetent individuals, and are a major cause of blindness associated with ocular trauma [3], [4]. Although less common, *Curvularia*, *Alternaria*, and *Penicillium* species also cause keratitis [3]. Globally, the world health organization estimates that 1.8 million people in developing nations are blinded annually from corneal ulcers; furthermore, in developing nations, up to 65% of total corneal ulcers are caused by fungal infection, with approximately 1 million cases occurring annually in Asia and Africa [5]–[7].

Treatment regimens for fungal keratitis are often ineffective, with up to 60% of fungal keratitis cases requiring corneal transplantation [3]. Given the limited treatment options, there is a pressing need to develop new treatment strategies. In this effort, we recently demonstrated that inhibitors of fungal anti-oxidative responses enhanced fungal clearance in vivo and improved disease outcome [8]. As iron is essential for the redox reactions of major fungal antioxidants, including thioredoxin-dependent peroxiredoxases [9], [10], and fungal iron acquisition mutants are more susceptible to oxidative stress [11], we hypothesized that targeting fungal iron acquisition may represent a potential new avenue for treatment of fungal infections.

Fungal iron acquisition primarily involves the production of hydroxamate-type siderophores that are secreted into the environment, bind iron with high affinity, and are then captured by specific siderophore receptors on the fungal cell membrane [12]. Specifically, the *A. fumigatus* siderophore biosynthesis pathway originates with the *sidA* gene, which encodes ornithine-*N^5^*-oxygenase, resulting in conversion of ornithine to *N^5^*-hydroxyornithine [13]. Utilizing this essential precursor, the siderophore biosynthesis pathway leads to either intracellular or extracellular siderophores. The *sidC* gene product is required for production of the intracellular siderophores, ferricrocin (FC) and hydroxyferricrocin (HFC), whereas the *sidF* and *sidD* gene products are required for production of the extracellular siderophores, fusarinine C (FusC) and triacetylfusarinine C (TAFC) [13]. The *sidG* gene product is required to generate TAFC from FusC [13], whereas both the *sidH* and *sidI* gene products are required to incorporate mevalonate into the structure of extracellular siderophores [14]. As mevalonate biosynthesis is dependent on HMG-CoA reductase and this enzyme is inhibited by statins, we hypothesized that statin-mediated inhibition of fungal HMG-CoA reductase may restrict fungal iron acquisition in vivo.

Fungal siderophores are secreted into mammalian tissues during infection where they compete with host iron sequestration defenses. Under homeostatic conditions, free iron is maintained at relatively low levels by iron-binding proteins such as transferrin and ferritin [15]. In addition, mucosal secretions contain high concentrations of lactoferrin, which binds iron, and lipocalin-1, which sequesters fungal siderophores [16], [17]. However, tissue damage during infection increases extracellular iron levels by releasing intracellular labile iron, ferritin, and heme-containing proteins [18]. Infection also stimulates both local and systemic immune defenses to counter microbial iron acquisition. Resident cells can secrete iron-sequestering proteins and chemotactic cytokines, which recruit neutrophils to the site of infection. Neutrophils also release pre-formed and *de novo* synthesized iron sequestering proteins such as lactoferrin, Lcn-2, and the hemoglobin binding protein haptoglobin [19]. Furthermore, neutrophil-mediated oxidation is likely to increase the microbial requirement for iron to fuel iron-dependent anti-oxidative defenses. Lastly, production of cytokines such as IL-6 and IL-22 can induce local and systemic synthesis of the peptide hormone hepcidin, which degrades the iron exporter ferroportin and traps iron inside host cells [20]–[22].

In the current study, we examined the role of host iron sequestration and fungal iron acquisition in a murine model of *Aspergillus* and *Fusarium* corneal infection. We show that Dectin-1 and IL-6 regulate expression of genes involved in iron sequestration and that fungal growth positively correlates with serum iron levels. Using mutant *A. fumigatus* and *F. oxysporum* strains, we also demonstrate that fungal transcriptional responses to low iron levels and mevalonic acid-dependent extracellular siderophore biosynthesis, but not intracellular siderophores or reductive iron assimilation, are essential for fungal growth *in vitro* and during infection. Lastly, using iron chelators, siderophore binding proteins, and siderophore biosynthesis inhibitors including statins we provide proof-of-concept that targeting fungal iron acquisition enhances fungal clearance from infected tissues and may represent a new avenue for treatment of fungal infections.

## Results

### Dectin-1 and IL-6 regulate local and systemic endogenous host iron-sequestration pathways

**Figure 1:**
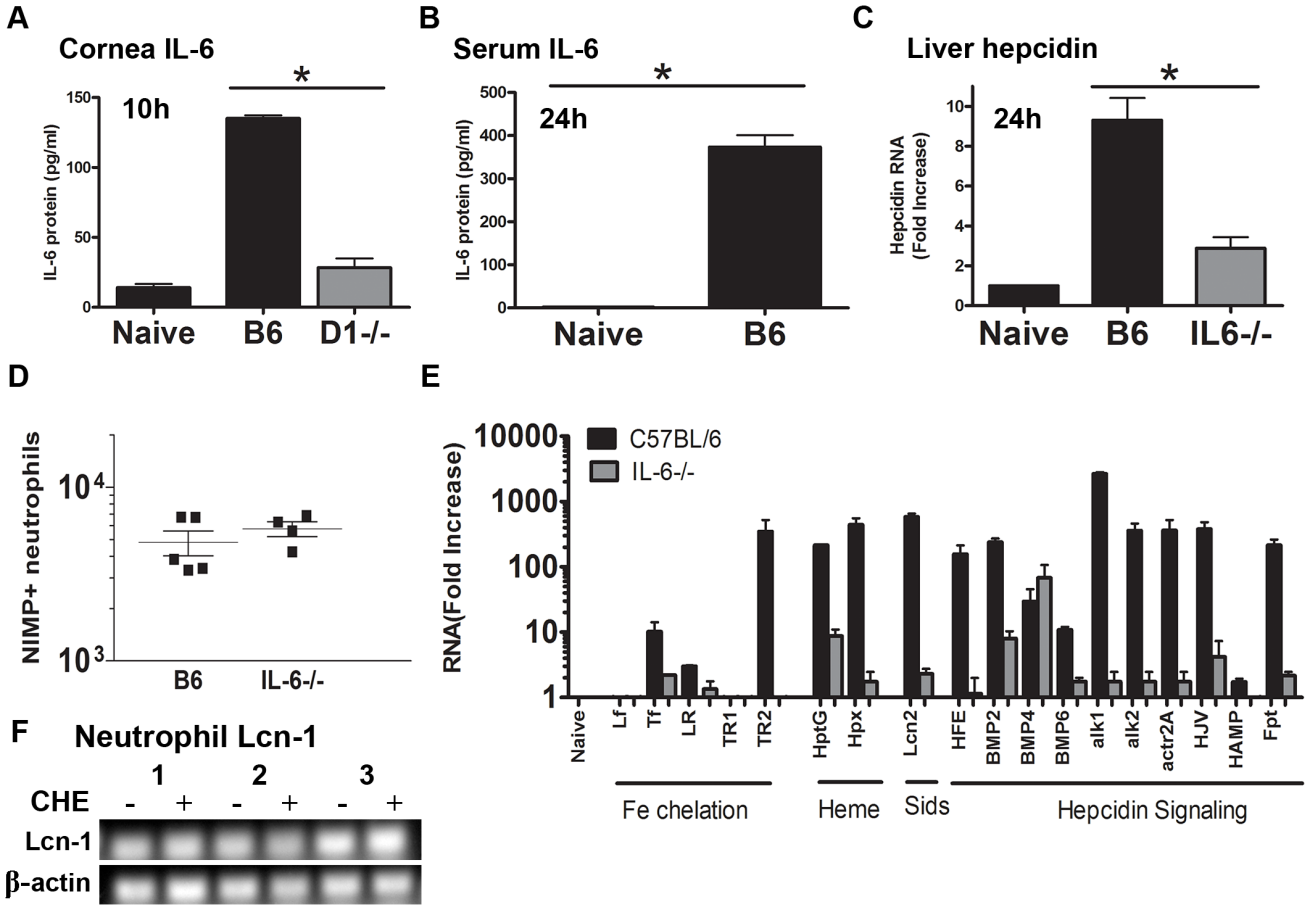
Expression of local and systemic iron-sequestration proteins in *Aspergillus fumigatus* infected corneas. **A.** IL-6 production in corneas of C57BL/6 and Dectin-1^−/−^ mice 10 h after infection with *A. fumigatus*. **B.** Serum IL-6 at 24 h post-infection was quantified by ELISA **C.** Total liver hepcidin gene expression was quantified using qPCR 24 h after corneal infection. **D.** Total neutrophil numbers in corneas of C57BL/6 and IL-6^−/−^ mice 24 h post-infection. Neutrophils were incubated with the Ly6G NIMP-R14 Ab, and examined by flow cytometry. (Data points represent individual corneas) **E.** RNA was extracted from corneas of infected mice 24 h post-infection, and genes encoding proteins involved in iron chelation, heme or siderophore sequestration, and hepcidin signaling were examined by qPCR. **F.** Lcn-1 gene expression in peripheral blood neutrophils from healthy volunteers after 2 h incubation with crude hyphal extract (CHE). (Data in panels A, B, C and E are mean +/− SD of 5 mice per group; Data in panel F are from three separate human donors). Abbreviations: **B6**-C57BL/6, **CHE**- crude Aspergillus hyphal extract, **D1**-Dectin-1, **Lf**-lactoferrin, **Tf**-transferrin, **LR**- lactoferrin receptor, **TR**- transferrin receptor, **HptG**- haptoglobin, **Hpx**- hemopexin, **Lcn**-lipocalin, **HFE**- human hemochromatosis protein, **BMP**-bone morphogenetic protein, **Alk**-activin receptor-like kinase, **Actr**-actin-related protein, **HJV**- hemojuvelin, **HAMP**-hepcidin, **Fpt**- ferroportin.

As we previously showed that Dectin-1 mediates neutrophil recruitment into the cornea during *Aspergillus* infection [23], and IL-6 is known to mediate hepatic hepcidin production [15], we examined the role of Dectin-1 and IL-6 in iron-sequestration during fungal infection. To determine if fungal infection of the cornea initiates an iron-sequestration response, we infected C57BL/6, Dectin-1^−/−^, and IL-6^−/−^ mice intrastromally with *A. fumigatus* dsRed conidia as described [8]. After 10 h, corneas were dissected and homogenized, and IL-6 was quantified by ELISA. **Figure 1A** shows increased IL-6 in the corneas of infected compared with naïve C57BL/6 mice at 10 h post-infection; however, IL-6 was not significantly elevated in Dectin-1^−/−^ corneas. Serum IL-6 was undetectable in naïve C57BL/6 mice; however, at 24 h post-infection serum IL-6 increased to 400ρg/ml in infected C57BL/6 mice (**Figure 1B**). As hepcidin expression is induced by IL-6 [15], we examined hepcidin expression in livers of infected C57BL/6 and IL-6^−/−^ mice at 24 h post-infection. **Figure 1C** shows that hepcidin expression was elevated 10-fold in infected compared with naïve C57BL/6 mice, whereas expression was significantly lower in infected IL-6^−/−^ mice.

During fungal infection of humans and mice, neutrophils comprise >95% of the cellular infiltrate in the cornea and are likely the predominant source of gene transcripts [23], [24]. Therefore, prior to examining de novo transcription of iron-sequestering genes during infection we first quantified the number of neutrophils infiltrating C57BL/6 and IL-6^−/−^ infected corneas by flow cytometry using the Ly6G NIMP-R14 monoclonal antibody. As shown in **Figure 1D**, there was no significant difference in neutrophil numbers between infected C57BL/6 and IL-6^−/−^ mice. RNA was then isolated from C57BL/6 and IL-6^−/−^ corneas at 24 h post-infection, and gene expression was measured by Q-PCR.

Compared with naïve C57BL/6 corneas, expression of the iron chelating protein transferrin (TF) was up-regulated 10-fold, transferrin receptor 2 (TR2) expression was up-regulated 400-fold, and lactoferrin receptor/intelectin-1 (LR) was elevated 3-fold in infected corneas (**Figure 1E**). In contrast, these genes were not elevated in infected IL-6^−/−^ corneas. Lactoferrin and transferrin receptor 1 expression were not elevated in C57BL/6 corneas, most likely due to these proteins being pre-formed in neutrophils [19].

During infection, lysed cells release heme, which microbes can utilize as a source of iron [18]. To restrict microbial access to heme, mammals produce hemopexin (Hpx) that binds to heme, and haptoglobin (HptG), which binds hemoglobin [15]. **Figure 1E** also shows that expression of Hpx and HptG in infected C57BL/6 mice is up-regulated 200-fold compared with naïve mice, whereas expression is <10-fold increased in infected IL-6^−/−^ mice.

In a low iron environment, microbial siderophores bind iron with high affinity and are subsequently internalized through siderophore transporters on the fungal cell membrane [12], [25]. Humans encode lipocalin-1 (Lcn-1) and lipocalin-2 (Lcn-2), whereas mice only express Lcn-2 [26]. Both Lcn-1 and Lcn-2 bind to bacterial siderophores, but only Lcn-1 binds fungal hydroxamate-type siderophores [17], [26]. We found that in infected C57BL/6 corneas, Lcn-2 expression is elevated 600-fold compared with 2-fold in IL-6^−/−^ mice (**Figure 1E**).

Although hepcidin is produced systemically by the liver, it is also produced by neutrophils and monocytes after stimulation with IL-6, or by iron-bound transferrin-mediated activation of the transferrin receptor 2/human hemochromatosis protein (HFE) complex [15]. Further, hepcidin induction is increased synergistically by bone-morphogenetic protein (BMP)-mediated activation and signaling through a hemojuvelin/BMP receptor complex [15]. **Figure 1E** shows that many of the proteins involved in non-inflammatory induction of hepcidin are up-regulated at 24 h post-infection, including HFE protein (200-fold), BMP2 (200-fold), BMP-4 (30-fold), BMP-6 (10-fold), the BMP receptors: alk1 (2000-fold) alk2 (300-fold), actr2A (300-fold), and hemojuvelin (300-fold). Interestingly, local hepcidin transcript is only 2-fold up-regulated at 24 h post-infection. In addition, ferroportin transcripts are up-regulated 250-fold during infection (**Figure 1E**), which may be a secondary response following hepcidin-mediated ferroportin degradation. **Figure 1E** shows that the transcription of all the hepcidin genes analyzed, except BMP-4, were significantly lower in infected IL-6^−/−^ mice compared to infected C57BL/6 mice.

Lcn-1 binds to many hydrophobic molecules including phospholipids at the air-fluid interface in tears [27]. However, Lcn-1 also binds to fungal hydroxamate-type siderophores [17]. Given that human neutrophils store the bacterial-siderophore binding protein Lcn-2 in secondary granules [19], we examined if human neutrophils also produce Lcn-1. Peripheral blood neutrophils (>95% purity) from three healthy human volunteers were incubated for 1 h in RPMI media in the presence or absence of *A. fumigatus* crude hyphal extract, and Lcn-1 expression was examined by Q-PCR. As shown in **Figure 1F**, Lcn-1 gene expression was detected in human neutrophils in the presence or absence of *Aspergillus* hyphal extract, indicating constitutive RNA expression. Taken together, results from this set of studies indicate that following fungal infection of the cornea, Dectin-1 dependent IL-6 production induces local and systemic host responses that limit microbial access to iron.

### Iron availability regulates hyphal growth and the severity of *A. fumigatus* infection

Given that fungal infection initiates an iron sequestration response, we next examined if iron availability regulates fungal growth during infection. Mice were injected intraperitoneally with 5 mg Fe-dextran (90 µmoles iron) or deferroxamine (5 mg), which is an iron-chelating xenosiderophore that is utilized by *A. fumigatus* [28]. Twenty four hours after the last injection, RFP-expressing *A. fumigatus* (Af-dsRed) conidia were injected into the corneal stroma of C57BL/6 mice [8], [23]. After 24 h, total serum iron was quantified in treatment and control groups.

**Figure 2:**
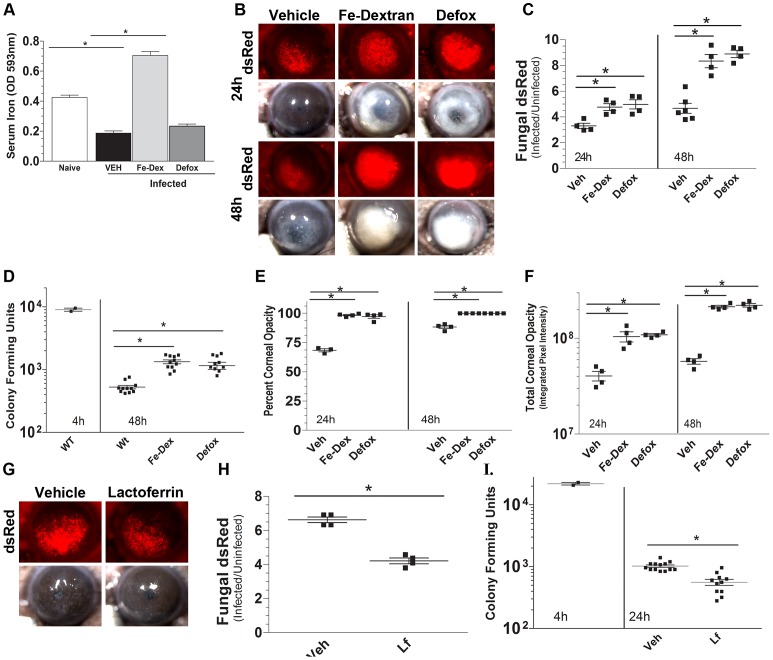
The effect of iron dextran, deferroxamine and lactoferrin on *A. fumigatus* corneal infection. **A.** Serum iron levels 24 h after corneal infection. C57BL/6 mice were pre-treated at day -2, and day-1 with I.P. injections of iron-dextran (Fe-Dextran) or deferroxamine (Defox), and serum iron levels were measured by spectrophotometry (data are mean +/− SD of 5 mice per group). **B.** Fungal growth (dsRed *A. fumigatus*) and corneal opacity in mice given Fe-Dextran or Defox. **C.** Metamorph image analyses of dsRed fluorescence. **D.** Colony forming units (CFU) per eye at 4 h and 48 h post-infection **E,F.** Metamorph image analyses of percent (**E**) and total (**F**) corneal opacification. **G–I:** Effect of lactoferrin on fungal growth. C57BL/6 mice were infected with *A. fumigatus* dsRed conidia, and given topical lactoferrin (10.4 µg) at 0 and 6 h post-infection. Corneas were examined after 24 h. **G:** representative images; **H.** image analysis of dsRed expression, and **I.** CFU per eye. Panels B and G show representative images, and data points in panels C–F, H, I represent individual corneas. All panels show representative data from one experiment except for panels D and I which show pooled data from repeat experiments. These experiments were repeated three times with similar results.

**Figure 2A** shows that serum iron levels in infected mice were reduced 2-fold compared with naïve mice, indicating systemic iron-sequestration during inflammation [15]. In contrast, mice given systemic Fe-dextran, but not deferroxamine had significantly elevated serum iron compared to vehicle-treated mice. Despite the difference in serum iron levels, **Figures 2B,C** show that whereas fungal mass (dsRed) increases over 48 h in vehicle-treated mice, this was significantly higher in Fe-dextran and deferroxamine-treated mice. Consistent with these data, **Figure 2D** shows that at 48 h post-infection, CFU were significantly higher in Fe-dextran and deferroxamine-treated mice compared with control, vehicle-treated mice. As filamentous fungi grow by hyphal extension and not cell division, the dsRed measure of fungal mass increases over time, whereas CFU decreases from the initial inoculum [15]. **Figures 2B, E, and F** show that corneal opacification was also increased in Fe-dextran and deferroxamine-treated mice compared to vehicle-treated mice, consistent with increased fungal growth. The increased fungal growth in deferroxamine treated mice is likely due to its xenosiderophore function, which can be used by *Aspergillus* for iron acquisition [28].

To examine the effect of limiting iron availability during infection, C57BL/6 corneas were infected with *A. fumigatus* dsRed conidia as described (12), and the iron chelating protein lactoferrin was added topically (10.4 µg in 8 µl) at 0 h and 6 h post-infection. As shown in **Figures 2G–I**, infected corneas given topical lactoferrin had significantly less fungal mass (dsRed) and CFU per eye at 24 h post-infection compared with those given vehicle alone. Taken together with results from Fe-dextran and deferroxamine-treated mice, these studies demonstrate that fungal growth in the cornea is dependent on increased free iron or bio-available iron.

### Siderophores and detection of low iron concentrations but not reductive iron assimilation is required for fungal growth during infection

Given that fungal growth during infection is enhanced by the exogenous xenosiderophore deferroxamine, we next examined the role of endogenous fungal siderophores using *A. fumigatus*, *F. oxysporum*, and *Alternaria brassicicola* iron acquisition mutants. The *A. fumigatus* Δ*sidA* mutant does not synthesize extracellular or intracellular siderophores [29], whereas the Δ*hapX* strain lacks the transcription factor HapX that is activated by low iron concentrations, and which regulates expression of genes involved in iron acquisition including siderophores and repression of iron-dependent pathways [30].

**Figure 3:**
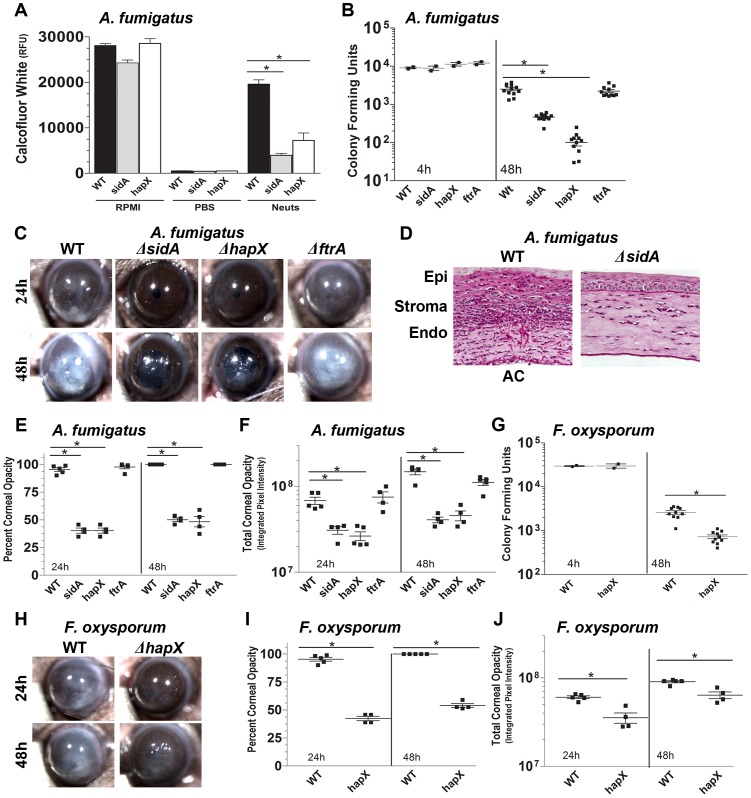
Susceptibility of *A. fumigatus* siderophore and iron acquisition mutants in fungal keratitis. **A.** Growth *A. fumigatus* Δ*sidA* and Δ*hapX* mutants after incubation with human neutrophils; fungal mass was quantified using calcofluor white and fluorometry. (Mean +/− SD of 5 replicate wells.) **B–F:** *A. fumigatus* corneal infection; **G–J** *F. oxysporum* corneal infection. **B:** CFU per eye of C57BL/6 mice 48 h after intrastromal infection with *A. fumigatus* Δ*sidA*, Δ*hapX*, or Δ*ftrA* mutants. **C.** Representative eyes showing corneal opacification at 24 h and 48 h. **D.** Representative corneal sections stained with periodic acid-schiff and hematoxylin (PASH). **E.** Quantification of percent corneal opacity and **F.** Total cornea opacity. **G–J.** C57BL/6 corneas were infected with *F. oxysporum* Δ*hapX* and the parent strain. **G.** CFU per eye at 4 h and 48 h post-infection **H.** Representative eyes showing corneal opacification. **I.** Quantification of percent corneal opacity **J.** Total cornea opacity. Data points represent individual corneas. All panels show representative data from one experiment except for panels B and G which show pooled data from repeat experiments. Similar results were found in three repeat experiments. Abbreviations: **Neuts**- neutrophils, **epi**-epithelium, **endo**-endothelium, **AC**-anterior chamber.

**Figure 3A** shows no significant difference in fungal growth in media alone between the parent (WT) strain and the Δ*sidA* and Δ*hapX* mutants, indicating that there is no effect of these mutations on fungal growth in the presence of an exogenous source of iron. We next examined the growth of the WT strain and the Δ*sidA* and Δ*hapX* mutants in the presence of neutrophils, which we recently showed kill hyphae by producing reactive oxygen species [8]. Conidia (spores; 12,500) were cultured 4–6 h in SDB media to allow germination and production of hyphae and incubated with 1×10^5^ neutrophils. In contrast to RPMI media alone in which all strains grew equally, in the presence of human neutrophils, growth of the Δ*sidA* and Δ*hapX* mutants was significantly less than the WT parental strain (**Figure 3A**), indicating that adaptation to iron starvation and siderophores, which requires SidA and HapX is essential for survival in the presence of neutrophils.

Consistent with a role for these genes in virulence, we found that at 48 h post-infection, corneas infected with the *A. fumigatus* mutant strains Δ*sidA* or Δ*hapX* had significantly lower CFU compared with the parent strain (**Figure 3B**). **Figure S1** shows that corneas infected with the complemented Δ*sidA* strain sidA**^R^** or the complemented Δ*hapX* strain hapX**^R^**, show no significant difference in opacification or CFU as the WT parental strain. In contrast, although the Δ*ftrA* mutant lacks a membrane-bound iron transport channel protein and is therefore deficient in cellular uptake of environmental iron [29], there was no significant difference in CFU in this mutant, indicating that this transporter protein is not essential for fungal growth *in vivo*. Similarly, **Figures 3C, E, and F** show that mice infected with the Δ*sidA* or Δ*hapX* mutants have significantly less corneal opacification than the parent *A. fumigatus* strain, whereas the Δ*ftrA* mutant was not significantly different. Histological analysis shows a pronounced cellular infiltrate and fungal hyphae in the corneas of mice infected with the parent strain, whereas no hyphae were detected in Δ*sidA* infected corneas, indicating that these mutants did not germinate in the cornea (**Figure 3D**). As with *A. fumigatus*, mice infected with the *F. oxysporum* Δ*hapX* strain also exhibit significantly lower CFU than the parent strain at 48 h post-infection (**Figure 3G**), and *A. brassicicola* siderophore mutants have significantly less CFU than the parent strain (**Figure S2**). **Figure 3H–J** demonstrate that mice infected with the *F. oxysporum* Δ*hapX* strain also exhibit significantly less corneal opacification compared to the parental strain. **Figure S1** shows that corneas infected with the *Fusarium* complemented Δ*hapX* strain hapX**^R^** show no significant phenotypic differences from the WT parental strain. Taken together, these data indicate that siderophores have a critical role in fungal growth during infection, whereas reductive iron assimilation is not essential.

### Extracellular siderophores are required for fungal infection

**Figure 4:**
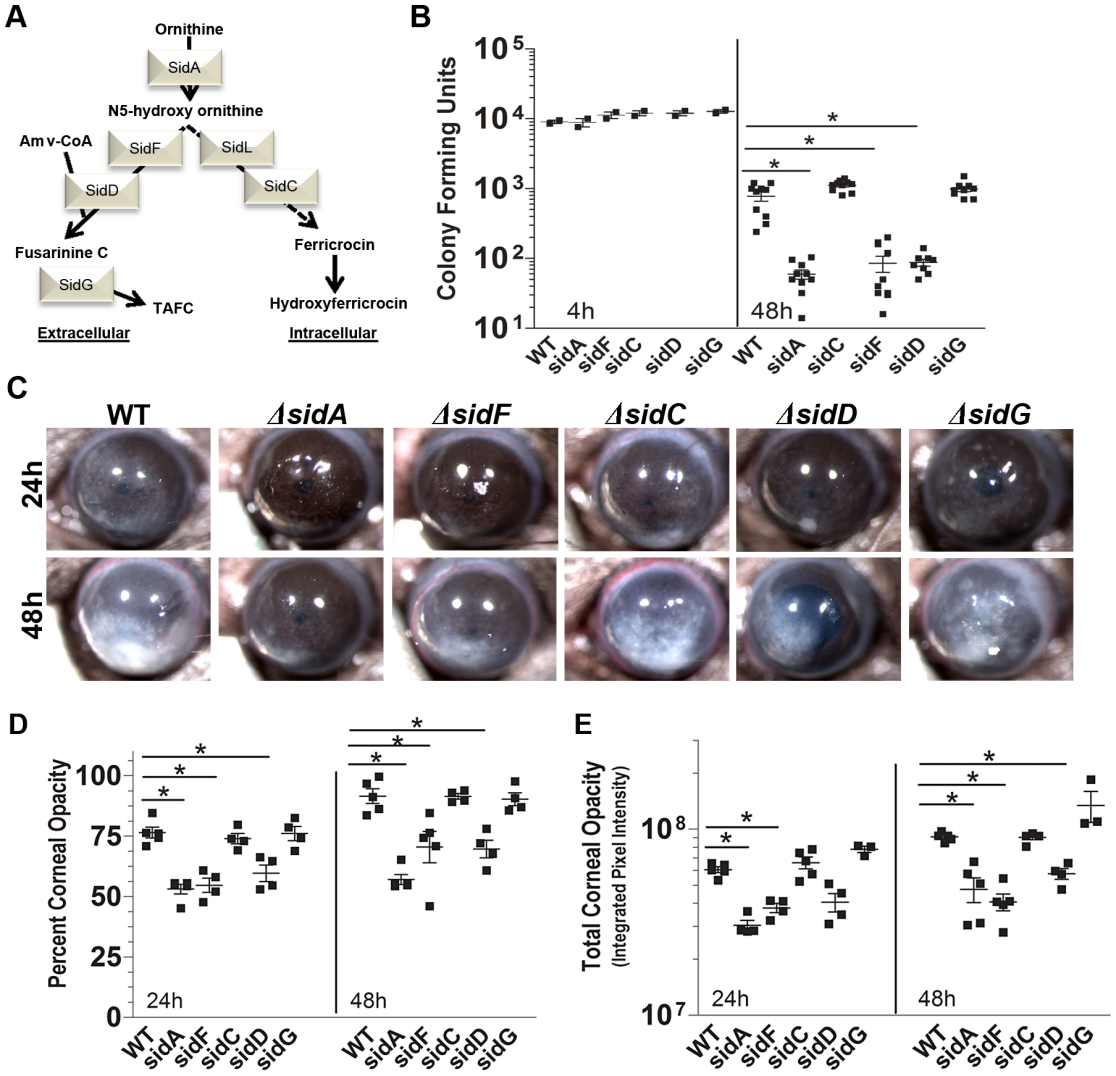
The role of intracellular and extracellular siderophores in *A. fumigatus* corneal infection. **A.** Biosynthesis of *A. fumigatus* siderophores. **B–E.** C57BL/6 mice infected intrastromally with *A. fumigatus* mutant strains Δ*sidA*, Δ*sidC*, Δ*sidF*, Δ*sidD*, or Δ*sidG*. **B.** CFU per eye at 4 h and 48 h post-infection. **C.** Representative eyes showing corneal opacity. **D.** Quantification of percent corneal opacity and **E.** total cornea opacity. B, D, E: Data points represent individual corneas. All panels show representative data from one experiment except for panel B which shows pooled data from repeat experiments. Similar results were found in 3 repeat experiments. Abbreviations: **Amv**- anhydromevalonyl; **TAFC:** tri-acetyl fusarinine C.

To determine the relative contribution of intracellular versus extracellular siderophores in fungal keratitis, corneas were infected with the Δ*sidF* and Δ*sidC* mutants. **Figure 4B** shows that CFU from corneas infected with the Δ*sidF* mutant that leads to extracellular siderophore production was significantly lower than those infected with WT *A. fumigatus*, having a similar CFU as Δ*sidA* mutants. In contrast, Δ*sidC* mutants that regulate production of intracellular siderophores were not significantly different from the parent strain. Consistent with this finding, corneas infected with Δ*sidF* had significantly lower cornea opacity area and intensity values compared with mice infected with WT *A. fumigatus*, whereas Δ*sidC* mutants were not significantly different (**Figure 4C–E**). Together, these findings indicate that extracellular, but not intracellular siderophores are essential for fungal growth in the cornea and development of keratitis.

To determine the relative contribution of the extracellular siderophores FusC and TAFC, we infected corneas with Δ*sidD* mutants, which do not produce extracellular siderophores, or with Δ*sidG* mutants, which produce FusC but not TAFC, and compared them with the parent *A. fumigatus* strain that produces both FusC and TAFC [13]. **Figure 4B** shows that mice infected with Δ*sidD* mutants had significantly lower CFU compared with WT *A. fumigatus*, whereas Δ*sidG* were not significantly different. Corneal opacification scores reflected the CFU data, with Δ*sidD* but not Δ*sidG* mutants having significantly less opacification than WT *A. fumigatus* (**Figure 4C–E**). These findings indicate that *sidD*-mediated synthesis of FusC rather than *sidG*-mediated synthesis of TAFC is necessary and sufficient to support fungal growth in vivo. **Figure S1** shows that corneas infected with the complemented strains sidF**^R^** and sidD**^R^** show no phenotypic differences from the WT parental strain. **Figure S2** shows that both intracellular and extracellular siderophore mutants of *Alternaria brassicicola* also have impaired growth during infection. Together, these data clearly demonstrate that extracellular siderophores are essential for both *Aspergillus* and *Alternaria* growth during tissue infection, and that even though TAFC is reportedly more stable [13], FusC production is sufficient to maintain fungal growth *in vivo*.

### Mevalonate incorporation into extracellular siderophores is required for fungal infection

**Figure 5:**
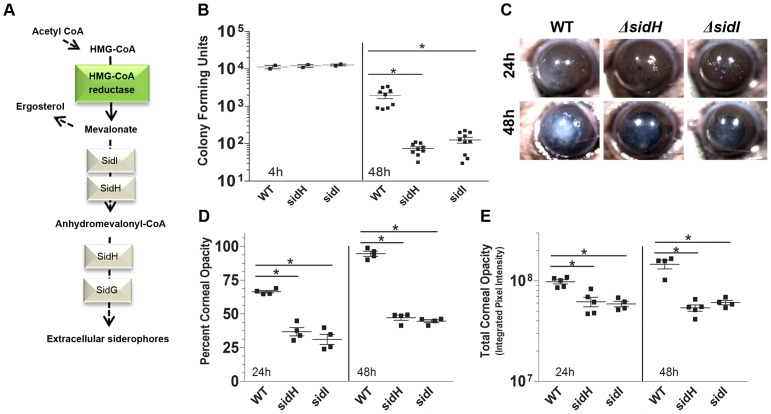
Role of mevalonate pathway for extracellular siderophores in *A. fumigatus* corneal infection. **A.** Mevalonate extracellular siderophore biosynthesis pathway. **B–E.** C57BL/6 mice infected intrastromally with *A. fumigatus* Δ*sidH* and Δ*sidI* mutant strains. **B.** CFU per eye at 4 h and 48 h post-infection. **C.** Representative eyes showing corneal opacity **D.** Quantification of percent corneal opacity and **E.** total corneal opacity. B, D, E: Data points represent individual corneas. All panels show representative data from one experiment except for panel B which shows pooled data from repeat experiments. Similar results were found in 3 repeat experiments. Abbreviations: **HMG-CoA**: 3-hydroxy-3-methyl-glutaryl-CoA.

Fungal extracellular siderophore biosynthesis requires HMG-CoA reductase-dependent synthesis of the precursor mevalonate [14]. The *Aspergillus* genes *sidI* and *sidH* encode a CoA-ligase and an enoyl-CoA-hydratase, respectively, which convert mevalonic acid to anhydromevalonyl CoA and incorporate this precursor through the sidF-D-G pathway into the structure of fusarinine C and TAFC (**Figure 5A**) [13], [14].

To determine if this pathway is essential for fungal growth during tissue infection, C57BL/6 mice were infected with *A. fumigatus* mutant strains Δ*sidH* and Δ*sidI* and examined as before. **Figure 5B** shows that mice infected with Δ*sidH* or Δ*sidI* exhibit significantly less CFU than mice infected with the WT strain, indicating that mevalonate incorporation into extracellular siderophores is essential for fungal growth during tissue infection. Further, mice infected with either Δ*sidH* or Δ*sidI* exhibit significantly less cornea opacity at all time-points compared to mice infected with WT *A. fumigatus* (**Figure 5C–E**). **Figure S1** shows that corneas infected with the complemented Δ*sidH* strain sidH**^R^** or the complemented Δ*sidI* strain sidI**^R^** show no significant difference in opacification or CFU as the WT parental strain.”

### Lipocalin-1 sequesters fungal siderophores and restricts fungal growth during infection

**Figure 6:**
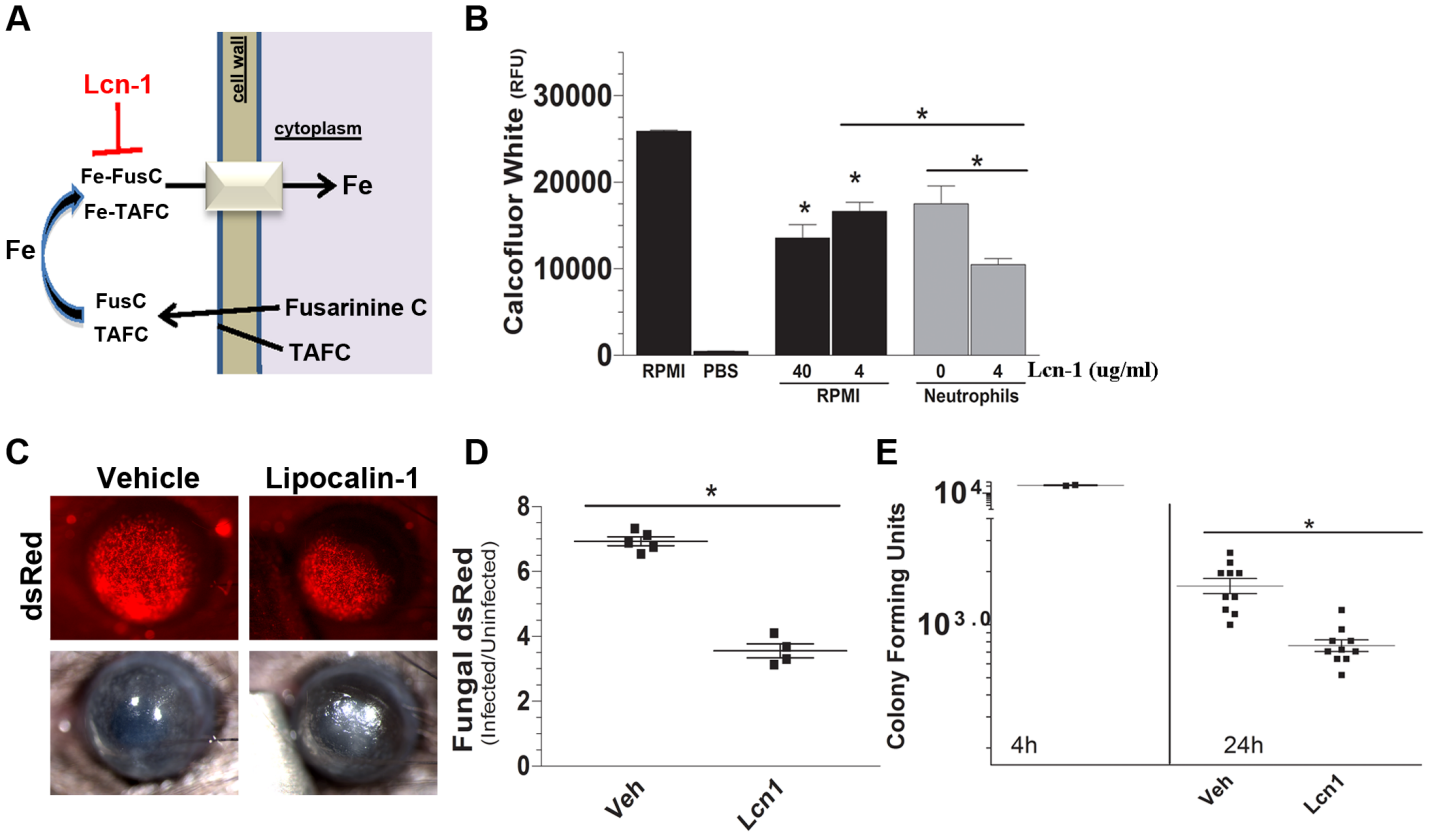
Effect of exogenous lipocalin-1 in *A. fumigatus* corneal infection. **A.** Pathway showing Lcn-1 sequestration of fungal siderophores. **B.** Growth of *A. fumigatus* incubated with recombinant human Lcn-1 in the absence (black bars) or presence (gray bars) of human neutrophils determined by calcofluor white binding and quantification using fluorometry (data are mean +/−SD of five replicate wells). **C–E:** C57BL/6 mice were given topical Lcn-1 (16 µg) at 0 and 6 h post-infection with *A. fumigatus* dsRed. **C.** Representative corneas; **D.** Metamorph image analysis showing fungal dsRed expression and **E.** CFU per eye. Data points represent individual corneas. All panels show representative data from one experiment except for panel E which shows pooled data from repeat experiments. Similar results were found in two repeat experiments. Abbreviations: **FusC**- fusarinine C, **TAFC**- tri-acetyl fusarinine C, **Lcn**-lipocalin.

Humans produce two lipocalins with siderophore binding activity [26]. Lipocalin-1 (Lcn-1) binds to a wide range of bacterial and fungal hydroxamate-type siderophores [17], whereas Lcn-2 binds catechol-type bacterial siderophores but not fungal siderophores [31], [32]. We therefore examined the role of Lcn-1 on *A. fumigatus* using the same assays as above. **Figure 6B** shows significantly less fungal growth incubated with 40 µg/ml or 4 µg/ml Lcn-1 than in RPMI alone, and that growth of *A. fumigatus* in the presence of neutrophils and 4 µg/ml Lcn-1 was significantly less than with neutrophils alone or Lcn-1 alone. **Figures 6C–E** show significantly less fungal dsRed and CFU in mice given topical Lcn-1 (16 µg/8 µl) at 0 h and 6 h after infection compared with infected mice not given Lcn-1. These findings indicate that topical Lcn-1 inhibits fungal growth *in vivo*, presumably by sequestering fungal siderophores.

### Topical simvastatin and deferiprone inhibit fungal infection

**Figure 7:**
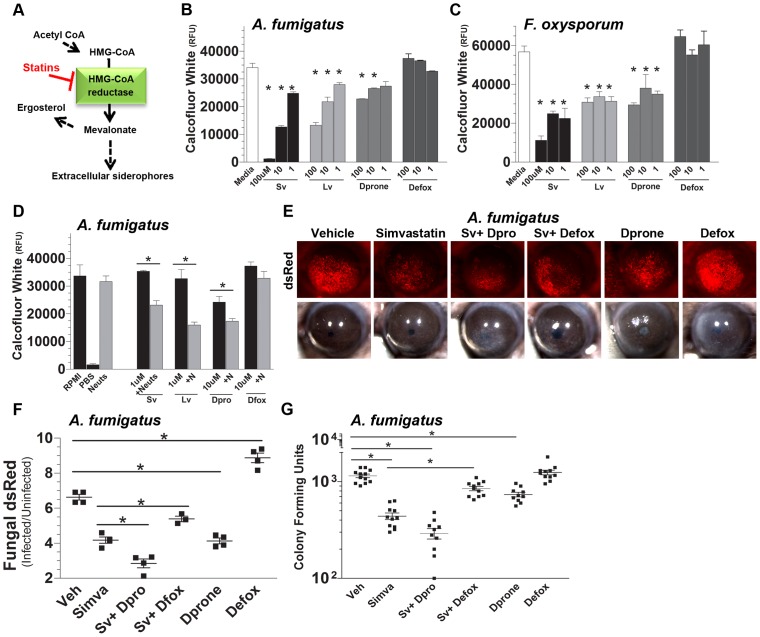
Effect of topical simvastatin and deferiprone on fungal infection. **A.** Statin targeting of fungal HMG-CoA reductase in siderophore biosynthesis. **B, C.** Effect of statins and iron chelators on growth of *A. fumigatus* and *F. oxysporum in vitro*. **B.** Simvastatin, lovastatin, deferiprone, or deferroxamine were added to growing cultures of *A. fumigatus* or **C.** *F. oxysporum* for 16 h, and hyphal growth was quantified by calcofluor white. **D.** Growth of *A. fumigatus* incubated with these compounds in the absence (black bars) or presence (gray bars) of human neutrophils by calcofluor white quantification (data are mean +/−SD of five replicate wells) **E.** C57BL/6 mice were infected with *A. fumigatus* and at 0 and 6 h post-infection 13.4 µg of simvastatin (Sv), deferiprone (11.1 µg), deferroxamine (52.5 µg), Sv+ deferiprone, or Sv+ deferroxamine was applied topically to infected corneas and eyes were imaged at 24 h post-infection. **F.** Metamorph image analysis was used to quantify fungal dsRed expression and **G.** eyes were homogenized for CFU analysis. B–D: data are mean +/−SD of five replicate wells; F,G: data points represent individual corneas. All panels show representative data from one experiment except for panel G which shows pooled data from repeat experiments. Similar results were found in three repeat experiments. Abbreviations: **HMG**- 3-hydroxy-3-methyl-glutaryl-CoA, **Sv**-simvastatin, **Lv**-lovastatin, **Dprone**- deferiprone, **Defox**-deferroxamine.

As shown in Figure 5, *Aspergillus* SidI and SidH proteins incorporate mevalonate into the structure of extracellular siderophores and are essential for fungal growth in the cornea. Also, HMG-CoA reductase is required for mevalonate production and can be targeted by statins to inhibit siderophore biosynthesis [14], [33] (**Figure 7A**). To determine the effect of blocking this pathway on fungal growth, *A. fumigatus* and *F. oxysporum* were incubated with the HMG-CoA reductase inhibitors simvastatin and lovastatin, or with the iron chelators deferiprone and deferroxamine.

As shown in **Figure 7B**, there was significantly less growth of *A. fumigatus* following 16 h incubation in SDB media with simvastatin, lovastatin and deferiprone, but not deferroxamine, thereby demonstrating a direct effect of statins and deferiprone on fungal growth. Similar results were obtained with *Fusarium oxysporum* (**Figure 7C**). Interestingly, a statin dependent dose curve was observed with *A. fumigatus* when exposed to simvastatin or lovastatin (**Figure 7B**), however, a dose curve was only observed when *Fusarium* was treated with simvastatin not lovastatin (**Figure 7C**). This observation likely reflects differences in the pharmacokinetics of different statins and their ability to penetrate into the fungal cytoplasm and inhibit HMG-CoA reductase of multiple fungal genera and species [33].

To ascertain if these agents enhance fungal sensitivity to killing by neutrophils, *A. fumigatus* conidia were cultured for 4–6 h in SDB media, washed, and incubated a further 16 h with human neutrophils in RPMI media containing simvastatin, lovastatin or deferiprone. As shown in **Figure 7D**, fungal growth was significantly less when incubated with neutrophils and simvastatin, lovastatin, and deferiprone compared with neutrophils alone, whereas there was no significant difference in the presence of deferroxamine. The inhibitory effect of 1 µM statins on fungal growth shown in panel B was not observed in this assay, most likely due to siderophore production during the 4–6 h growth in the absence of statins.

To ascertain if statins can restrict fungal growth during infection, mice were infected intrastromally with *A. fumigatus*, and given topical simvastatin, deferiprone, or deferroxamine at the time of infection and after 6 h. At 24 h post-infection, mice eyes were imaged for corneal opacity and fungal dsRed, and processed for fungal CFU. Importantly, unlike prior experiments, which examined CFU at the 48 h time-point, at 24 h CFU in infected eyes does not decrease unless treated with anti-microbial agents. Therefore, in this 24 h assay and unlike prior experiments, vehicle-treated CFU do not decrease but instead represent the maximum fungal CFU value per assay. As shown in **Figure 7E and F**, mice treated with simvastatin or deferiprone exhibited significantly less fungal mass compared to vehicle-treated mice, which was further decreased when given both compounds. Conversely, mice given simvastatin together with deferroxamine had significantly higher fungal mass than mice given simvastatin alone, indicating that exogenous deferroxamine counters the inhibitory activity of statins. **Figure 7G** shows similar responses when CFU were measured 24 h post infection, with significantly less CFU in mice given simvastatin, deferiprone or both, and partial reversal of the inhibitory effect of simvastatin when mice also received topical deferroxamine, indicating that simvastatin is targeting siderophore biosynthesis in addition to ergosterol synthesis *in vivo*. Mice treated with deferroxamine alone showed elevated fungal mass (**Figure 7E**) compared with vehicle-treated mice, but at this time point, CFU values were not significantly different from vehicle-treated mice (**Figure 7F**). Taken together, these findings clearly demonstrate that topical statins and iron chelation can block fungal growth during infection.

## Discussion

Previous work in our laboratory demonstrated that fungal anti-oxidative responses are essential for survival during tissue infection, and that fungal growth can be inhibited in vivo by targeting fungal thioredoxin [8]. As thioredoxin-regulated peroxiredoxases, catalases, and other antioxidants require iron to quench reactive oxidants [9], [34], the current study examined the role of host iron sequestration and fungal iron acquisition during infection. In examining endogenous iron levels, we found a two-fold reduction in serum iron levels following fungal infection which correlated with elevated expression of iron-chelating proteins, heme-binding and siderophore-sequestering proteins in infected corneas, and systemic induction of hepatic hepcidin. Expression of these iron-sequestering proteins was dependent on Dectin-1, which we and others showed is important in recognizing β-glucan on germinating conidia [23], [35], [36]. Expression of these proteins was also dependent on IL-6, which has been shown to induce liver hepcidin and reduce systemic iron levels [15].

**Figure 8:**
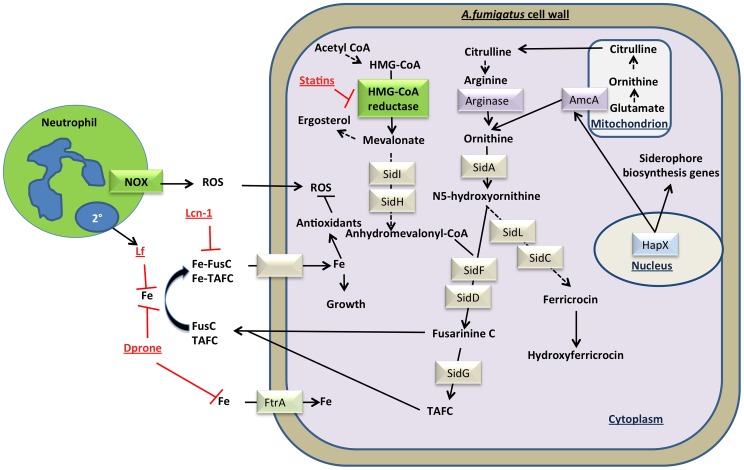
The battle for iron between the mammalian host and fungi. **Defox**- deferroxamine, **Dprone**- deferiprone, **Fe**-iron, **Fus C**- fusarinine C, **HMG-CoA**- 3-hydroxy-3methylglutaryl-coenzyme, **Lcn-1**- lipocalin 1, **Lf**- lactoferrin, **NOX**- nicotinamide adenine dinucleotide phosphate oxidase, **ROS**-reactive oxygen species, **TAFC**- triacetyl fusarinine C.

In this iron-restricted environment, we show that fungal siderophores are essential for microbial growth and survival. This notion is supported by our findings that siderophore mutants are unable to grow in the cornea, and that exogenous iron chelators or inhibitors of fungal siderophore biosynthesis impair fungal growth both in vitro when incubated with human neutrophils, and *in vivo* in a murine model of fungal infection. Given that the siderophore biosynthesis pathway is highly conserved in filamentous fungi [12], our findings are very likely relevant to fungal infections of other tissues in addition to other fungal pathogens. **Figure 8** illustrates the fungal siderophore biosynthesis pathway and highlights the key findings in this study.

Firstly, we identified that both *Aspergillus* and *Fusarium* require the transcription factor HapX to sense and respond transcriptionally to low iron levels and survive during infection. Further, we show that *A. fumigatus* strains Δ*sidF*, Δ*sidD*, Δ*sidH*, or Δ*sidI*, that do not express the extracellular siderophores are attenuated during infection of the cornea, whereas based on the absence of a phenotype with Δ*sidC* mutants, there is no apparent role for intracellular siderophores. We show that production of extracellular siderophores is also required for infection with *Alternaria*. These findings are consistent with an essential role for HapX and extracellular siderophores during experimental *A. fumigatus* lung infection [11], [14], [30], the requirement of HapX for *Fusarium* infection of tomato plants and immunosuppressed mice [37], and the requirement of siderophores for *Alternaria* infection in maize [38]. Interestingly, iron starvation also activates the transcription factor AcuM, which increases HapX expression and down-regulates the iron-repressing transcription factor SreA, resulting in fungal iron acquisition [39]. The role of AcuM and SreA during corneal infection has yet to be determined.

In the present study, we also examined the potential for statins to inhibit fungal HMG-CoA reductase, which is required for mevalonic acid production and extracellular siderophore biosynthesis [14]. Consistent with a report on pulmonary aspergillosis [14], we used mutants to demonstrate that SidI and SidH-dependent mevalonic acid incorporation into extracellular siderophores is essential for infection of the cornea. Further, we show that simvastatin and lovastatin inhibit fungal growth *in vitro*, and also enhance growth inhibition by human neutrophils. Consistent with the difference in statin activity in treating hypercholesterolemia [40], simvastatin exhibited 10-fold greater inhibitory activity than lovastatin in restricting fungal growth *in vitro*. During infection, topical application of simvastatin inhibited fungal growth that was only partially reversed by exogenous siderophores, indicating that statins function in vivo by inhibiting both fungal siderophore and ergosterol biosynthesis. The fungicidal activity of statins has been reported for *Aspergillus*, *Fusarium*, *Mucorales*, and *Candida* [41]–[43]. However, to our knowledge, this is the first study to clearly demonstrate a therapeutic effect of statins in an experimental fungal infection. In contrast to systemic statin treatment, topical application is likely to have minimal risk of side effects [33]. Future studies will examine if statins can restrict fungal growth at later stages of infection.

In addition to showing inhibition of siderophore biosynthesis, the current study demonstrates that exogenous lipocalin-1 impairs fungal growth in the presence of human neutrophils and during infection. This finding is consistent with the reported role for Lcn-1 in sequestering fungal siderophores, including TAFC [17]. The affinity of Lcn-1 for fungal siderophores is similar to that of fungal siderophore receptors [17], and therefore at high concentrations Lcn-1 could sequester siderophores from fungal receptors. Further, it is likely that Lcn-1-siderophore complexes are internalized through the lipocalin-interacting membrane receptor (LIMR) resulting in siderophore degradation [44], [45]. Endogenous Lcn-1 is abundant in human tears (3 mg/ml), nasal mucosa, and tracheal secretions where it can function prophylactically to prevent mucosal fungal infections [17], [46]. However, as we now show that human neutrophils express Lcn-1, it is possible that Lcn-1 also has a protective role during active infection.

In addition to targeting siderophores, we showed that reducing local tissue iron concentrations by topical application of the iron chelating protein lactoferrin restricts fungal growth *in vivo*. This finding is consistent with the reported role for lactoferrin in blocking conidia germination *in vitro* by human neutrophils [47], and suggests that *in vivo*, neutrophil-derived lactoferrin restricts the growth of conidia and hyphae by binding free iron. Given that siderophores exhibit a higher affinity for iron than lactoferrin [12], it is likely that fungal siderophores can acquire iron from lactoferrin during infection; however, the rate of siderophore iron acquisition in a lactoferrin-rich environment is likely slower than in the absence of lactoferrin given the scarcity of free iron or iron-bound to lower affinity biomolecules.

Similarly, we showed that the iron chelator deferiprone sensitizes fungi to human neutrophils and blocks fungal infection. However, deferiprone is also a very small molecule (MW = 140 g/mole), approximately 600-fold smaller than lactoferrin, and is therefore released from tissues more readily than lactoferrin, resulting in both iron sequestration and depletion from infected tissues [48]. Deferiprone has been used effectively and safely to lower iron levels in patients with hemochromatosis [48], and although widely utilized in Europe, it is not currently licensed in the USA. Importantly, deferiprone, unlike deferroxamine, is not a xenosiderophore [28] and is not associated with an increased risk of fungal and bacterial infections [49]–[57].

Iron chelators have been used to treat *Aspergillus* and *Rhizopus* infections in mice [57]–[60], and a clinical trial examined the potential of the iron chelator deferasirox to enhance the efficacy of liposomal amphotericin B to treat mucormycosis (the DEFEAT Mucor study). However, the trial was unsuccessful due to an unexpected increased risk of death in patients receiving deferasirox adjunct therapy [61]. The DEFEAT Mucor study exhibited a limited sample size and imbalanced stratification of the sickest patients into the deferasirox treatment group and may not accurately reflect the potential of deferasirox to treat mucormycosis. However, systemic deferasirox treatment does cause side effects that include agranulocytosis and nephrotoxicity [62]. In the current study, we demonstrated that local (topical) application of iron chelators is both effective in inhibiting fungal growth and preventing corneal disease. As local administration is highly unlikely to cause systemic side effects, clinical studies using topical iron chelators and fungal iron acquisition inhibitors are unlikely to cause adverse reactions, especially if combination therapies targeting iron acquisition can use low drug concentrations. This approach could be used to treat fungal infections not only in the cornea, but also in other tissues that could be treated topically such as the tongue, skin, and nails.

In conclusion, we have identified host iron sequestration and fungal siderophore biosynthesis as essential mediators of fungal growth during infection. One approach to exploiting these findings is to chelate local iron at the infectious site utilizing deferiprone or lactoferrin. A second approach is to inhibit the ability of fungi to acquire iron utilizing the siderophore-binding protein Lcn-1 or the siderophore biosynthesis inhibitor simvastatin. However, the most efficacious strategy would likely involve the combination of iron chelation and inhibition of siderophore biosynthesis. In this study, we provide proof-of-concept that dual treatment with deferiprone and simvastatin further restricts fungal growth during infection. As both deferiprone and simvastatin, have a long history of safe use in patients, it is possible that these agents can be successfully utilized to treat a broad range of fungal infections.

## Materials and Methods

### Use and source of animals

All animals were treated in accordance with the guidelines provided in the Association for Research in Vision and Ophthalmology ARVO statement for the Use of Animals in Ophthalmic and Vision Research, and were approved by Case Western Reserve University IACUC. C57BL/6 mice (6–12 wk old) and IL-6^−/−^ mice on a C57BL/6 background were from the Jackson Laboratory (Bar Harbor, ME), Dectin-1^−/−^ mice were kindly provided by Dr. Yoichiro Iwakura (University of Tokyo; Tokyo, Japan).

### Fungal strains and growth conditions

**Table 1** lists the genotype and phenotype of all strains utilized in this study. *Aspergillus fumigatus* was cultured on Vogel's minimal media (VMM) +2% agar and *Fusarium oxysporum* lycopersici was cultured on potato dextrose agar (PDA). *Alternaria brassicicola* was cultured in complete media as described previously [38]. All solid media used in this study were supplemented with 10 mM FeSO_4_ to enhance conidia production by siderophore mutants. For neutrophil-fungus incubation assays, all fungi were grown in RPMI media w/o FeSO_4_ supplementation. The *Alternaria brassicicola* Δ*nps2*, Δ*nps6*, and Δ*nps2/6* strains were kindly provided by Dr. B. Gillian Turgeon (Cornell University, Ithaca, NY).

**Table 1:** Fungal strains utilized in this study.

Strain	Genotype	Phenotype
***A. fumigatus***		
**Af-dsRed ** **[23]**	Af293.1- Δ*pyrG1*::gpdA::dsRed::pyrG	dsRed Fluorescence
**ATCC 46645**		WT
**Δ** ***sidA*** **** **[29]**	ATCC 46645- sidA::hph	No intra or extracellular siderophores
***sidA^R^*** **** **[29]**	ΔsidA- sidA::sidA	Complemented strain
**Δ** ***sidC*** **** **[11]**	ATCC 46645- sidC::hph	No intracellular siderophores
**Δ** ***sidD*** **** **[11]**	ATCC 46645- sidD::hph	No fusarinine C or TAFC
***sidD^R^*** **** **[11]**	ΔsidD- sidD::sidD	Complemented strain
**Δ** ***sidF*** **** **[11]**	ATCC 46645- sidF::hph	No extracellular siderophores
***sidF^R^*** **** **[11]**	ΔsidF- sidF:: sidF	Complemented strain
**Δ** ***sidG*** **** **[11]**	ATCC 46645- sidG::hph	No TAFC
**Δ** ***sidH*** **** **[14]**	ATCC 46645- sidH::hph	No extracellular siderophores
***sidH^R^*** **** **[14]**	ΔsidH- sidH::sidH	Complemented strain
**Δ** ***sidI*** **** **[14]**	ATCC 46645- sidI::hph	No extracellular siderophores
***sidI^R^*** **** **[14]**	ΔsidI- sidI::sidI	Complemented strain
**Δ** ***hapX*** **** **[30]**	ATCC 46645- hapX::hph	No hapX
***hapX^R^*** **** **[30]**	ΔhapX- hapX::hapX	Complemented strain
**Δ** ***ftrA*** **** **[29]**	ATCC 46645- ftrA::hph	No reductive iron assimilation
***F. oxysporum***		
**FoxL- 4287 ** **[37]**		WT
**Δ** ***hapX*** **** **[37]**	4287- hapX::hph	No hapX
***hapX^R^*** **** **[37]**	ΔhapX- hapX::hapX	Complemented strain
***A. brassicicola***		
**WT-Tf383**		WT
**Δ** ***nps2*** **** **[38]**	Tf383- nps2::hph	No intracellular siderophores
**Δ** ***nps6*** **** **[38]**	Tf383- nps6::hph	No extracellular siderophores
**Δ** ***nps2/6*** **** **[38]**	Tf383- nps2::hph; nps6::hph	No intra or extracellular siderophores

### Mouse model of *Aspergillus* and *Fusarium* keratitis

*Aspergillus* and *Fusarium* strains were cultured as described above for 2–3 days, and fresh conidia were disrupted with a bacterial L-loop, harvested in 5 ml PBS, and filtered through sterile PBS-soaked cotton gauze in a 10 ml syringe to obtain pure conidial suspensions. Conidia were quantified using a hemocytometer and adjusted in PBS to a final stock solution of 15–20,000 conidia/µl. Mice were anaesthetized with 1.25% 2, 2, 2-tri-bromoethanol in PBS. The corneal epithelium was abraded using a 30-gauge needle, through which a 2 µl injection containing conidia was released into the corneal stroma using a 33-gauge Hamilton syringe. Mice were examined daily under a stereomicroscope for corneal opacification, and quantified by image analysis using Metamorph software as described [8],[23]. At each time point, animals were euthanized by CO_2_ asphyxiation, and eyes were either placed in 10% formalin and embedded in paraffin, sectioned at 5 µm intervals and stained with periodic acid Schiff and Hematoxylin (PASH), or were placed in 1 ml of sterile PBS, homogenized and colony forming units (CFU) were quantified by manual count.

### Topical and systemic drug delivery

Compounds were suspended in a commercial eye drop formulation (Alcon laboratories) or in PBS, and 8 µl was applied topically at 0 h and 6 h post-infection. Lactoferrin (1.25 mg/ml), deferiprone (10 mM), deferroxamine (10 mM), and simvastatin (4 mM) were purchased from Sigma Aldrich (St.Louis, MO). *E.coli*-expressed recombinant human lipocalin-1 was purified as described previously [17] and applied topically at 2 mg/ml. Iron-dextran and deferroxamine were purchased from Sigma and administered systemically to mice by daily intraperitoneal injections of 5 mg starting at day -2 until mice were euthanized. All animals were bred under specific pathogen-free conditions and maintained according to institutional guidelines.

### Quantification of *Aspergillus* fungal mass and colony forming units (CFUs)

Growth of the RFP expressing *A. fumigatus* strain in the cornea was detected by fluorescent microscopy and quantified by Metamorph image analysis [8], [23]. For assessment of fungal viability, whole eyes were homogenized under sterile conditions in 1 ml PBS, using the Mixer Mill MM300 (Retsch) at 33 Hz for 4 min. Subsequently, 100 µl aliquots were plated onto bacteriologic-grade Sabouraud dextrose agar plates, incubated for 24 h at 37°C (*Aspergillus*) or at 30°C (*Fusarium* and *Alternaria*), and the number of CFU/eye was determined by direct counting. The weight of the whole eye is consistent from one mouse to the next regardless of infection, and as we homogenize the entire eye and not just the cornea, we calculate CFU from the entire eye not just a representative sample. Fungal dsRed and CFU analysis do not have a linear correlation as hyphae of varying lengths show differences in dsRed fluorescence, but are still counted as a single CFU. Also, as homogenization can potentially damage branched hyphae, which may be more abundant in wild type compared with mutants, we may be underestimating the difference in CFUs between strains. All CFU graphs show pooled data from at least three repeat experiments.

### Quantification of IL-6 protein in mouse corneas and serum

Corneas were homogenized in 150 µl reagent diluent (R & D Systems, Minneapolis, MN) using the Retsch MM 300 ball miller at 33 Hz for 4 min (Qiagen). Mouse serum was obtained as described below and assayed directly. IL-6 protein was quantified using a mouse IL-6 ELISA kit as per manufacturer's instructions (R & D Systems, Minneapolis, MN).

### Quantification of neutrophils in mouse corneas

Corneas were dissected, cut into 8 small fragments, and incubated in 80 units of collaganese (Sigma-Aldrich) for 1–2 h. The cell suspensions were filtered, centrifuged at 300*g for 5 min at 4°C and washed in FACS buffer (PBS+1% FBS+0.5% Na azide). Cells were then incubated with anti-mouse CD16/32 antibody (Fc block, clone 93, eBioscience) for 10 min followed immediately by incubation with biotinylated rat anti-mouse NIMP-R14 or isotype-control for 45 min. Cells were washed and incubated with streptavidin-PE-Cy7 for 30 min in the dark. Cell suspensions were then analyzed utilizing a C6 Accuri flow cytometer with gates set based on isotype controls.

### Quantitative PCR of infected corneas

C57BL/6 mice and IL-6^−/−^ mice were infected with *A. fumigatus* strain Af-dsRed as described above. At 24 h mice were sacrificed, corneas were excised, suspended in tissue lysis buffer (Qiagen, Valencia, CA) and homogenized using the Mixer Mill MM300 (Retsch) at 33 Hz for 2 min. Subsequently, RNA was extracted from samples using RNeasy mini kit according to the manufacturer's directions (Qiagen, Valencia, CA). Samples with a 260/280 (RNA∶protein) ratio of 2.0 were used to generate cDNA using the superscript first strand synthesis system (Life technologies, Grand Island, NY) using standard methods. Real Time PCR was performed on the cDNA samples using the SYBR green system (Applied Biosystems, Carlsbad, CA). All primers used in this study are listed in **Table 2** and were synthesized by Integrated DNA technologies (San Diego, CA). Fold change with respect to naïve uninfected corneas was calculated using the 2^−ΔΔct^ method. Data are therefore presented as fold increases of relative gene expression (log (RQ)). RT-PCR samples were also analyzed by 2% agarose gel electrophoresis.

**Table 2:** Primers sequences and protein function.

Primer	Source (PB ID)	Sequence	Protein Function
**Iron Chelation**			
Lactoferrin	141803548b2	CCGCTCAGTTGTGTCAAGAAA	Binds Fe at low pH
		CATGGCATCAGCTCTGTTTGT	
Transferrin	118129942b1	GCTGTCCCTGACAAAACGGT	Binds Fe at neutral pH
		GGTATTCTCGTGCTCTGACAC	
Tf receptor 1	291045184b1	GATCAAGCCAGATCAGCATTCT	Internalizes transferrin
		GTGTATGACAATGGTTCCCCAC	
Tf receptor 2	113204629b1	CGTTGGGGTCTACTTCGGAGA	Internalizes transferrin
		AGATGGTCTGAGAGGGTCTTG	
Lf receptor/intelectin 1	118130045b1	CAGCACTTGGGACATAATCTGT	Internalizes lactoferrin
		TCCTTCTCCGTATTTCACTGGG	
Lipocalin-1- Human	32455234b1	ATGTGTCAGGGACGTGGTATC	Binds fungal siderophores
		CCGATTCCAGATTCATCTCAGG	
**Heme Binding Protein**			
Haptoglobin	254910958b1	GCACTTGGTTCGCTATCGCT	Binds hemoglobin
		GCCCGTAGTCTGTAGAACTGT	
Hemopexin	160358828b1	AGCAGTGGCGCTAAATATCCT	Binds heme
		CAACTCTCCCGTTGGCAGTA	
**Siderophore Binding**			
Lipocalin-1	32455234b	ATGTGTCAGGGACGTGGTATC	Binds fungal siderophores
		CCGATTCCAGATTCATCTCAGG	
Lipocalin-2	34328048b1	GCAGGTGGTACGTTGTGGG	Binds bacterial siderophores
		CTCTTGTAGCTCATAGATGGTGC	
**Hepcidin Signaling**			
Interleukin 6	13624310b1	CTGCAAGAGACTTCCATCCAG	Induces HAMP* synthesis
		AGTGGTATAGACAGGTCTGTTGG	
HFE	145966687b1	CGGGCTGCCTTTGTTTGAG	Signal transduction→HAMP
		CTGGCTTGAGGTTTGCTCC	
BMP-2	71896668b1	TCTTCCGGGAACAGATACAGG	Amplifies HAMP synthesis
		TGGTGTCCAATAGTCTGGTCA	
BMP-4	121949822b1	ATTCCTGGTAACCGAATGCTG	“
		CCGGTCTCAGGTATCAAACTAGC	
BMP6	118131176b1	GCGGGAGATGCAAAAGGAGAT	“
		ATTGGACAGGGCGTTGTAGAG	
alk1- BMPR type 1a	133891829b1	TGGCACTGGTATGAAATCAGAC	BMP* receptor
		CAAGGTATCCTCTGGTGCTAAAG	
alk2-BMPR type 1b	41053831b1	CCTCGGCCCAAGATCCTAC	“
		CCTAGACATCCAGAGGTGACA	
actrIIA-BMPR type 2	145966831b1	GTGTTATGGTCTGTGGGAGAAAT	“
		AAAGCGGTACGTTCCATTCTG	
Hemojuvelin	166295197b1	ATGGGCCAGTCCCCTAGTC	BMPR signaling
		CAGCGGAGGATCTTGCACT	
Hepcidin	Blood 2010 [63]	CTGAGCAGCACCACCTATCTC	Degrades ferroportin
		TGGCTCTAGGCTATGTTTTGC	
Ferroportin	124248584b1	TGGAACTCTATGGAAACAGCCT	Exports iron from cells
		TGGCATTCTTATCCACCCAGT	
**Housekeeping gene**			
β-actin- Mouse	6671509a1	GGCTGTATTCCCCTCCATCG	Housekeeping gene
		CCAGTTGGTAACAATGCCATGT	
β-actin - Human	144922730c1	GTCTGCCTTGGTAGTGGATAATG	Housekeeping gene
		TCGAGGACGCCCTATCATGG	

* BMP: bone morphogenetic protein, HAMP: hepcidin, HFE: human hemochromatosis protein, Tf: transferrin.

### Quantification of iron content in mouse serum

Whole blood was obtained from mice by retro-orbital bleeding, and serum was recovered following blood coagulation. An iron assay kit (ABCAM, Cambridge, MA) was subsequently used to quantify Fe^2+^ and Fe^3+^ in the serum using manufacturer's instructions. Briefly, 25 µl of serum was added to 75 µl iron assay buffer and 5 µl iron reducer, which reduces Fe^3+^ to Fe^2+^. Next, 100 µl of the iron-probe solution was added yielding a Fe^2+^-ferene S complex that absorbs light at 593 nm. Spectrophotometry was used to detect absorbance at this wavelength.

### In vitro human neutrophil∶hyphae growth inhibition assay

Human neutrophils were isolated from normal, healthy donors using Ficoll-Paque Plus (GE) density centrifugation as described [8]. Isolated conidia from each *A. fumigatus* mutant were cultured in 200 µl SDA media (12,500/well) in black-wall 96 well plates with an optically clear bottom (CoStar 3720) until early germ tubes were observed (4–6 h). Wells were washed twice with sterile ddH_2_O and incubated 16 h with either RPMI media (+ Control), PBS (− Control), or human peripheral blood neutrophils suspended in RPMI at 0.5–1*10^5^/well, which we know does not inhibit fungal growth [8]. After 16 h incubation, plates were washed and stained with 50 µl calcofluor white stain, which binds chitin (Fluka 18909) for 5 min in the dark. Subsequently, plates were washed three times with ddH_2_O and quantified by fluorometry (360/440 nm; Synergy HT; Biotek). In assays where no neutrophils were added, fungi were incubated in SDA media alone with or without inhibitors. Fungi cultured in 96-well plates exhibit maximal growth by 16 h; therefore, this assay measures only relative decreases in fungal growth.

### Statistical analysis

Statistical analysis was performed for each experiment using one way ANOVA with a Tukey post-hoc analysis using Prism software (GraphPad Software Inc, La Jolla, CA). A p value<0.05 was considered significant.

### Ethics statement

All animals were treated in accordance with the guidelines provided in the Association for Research in Vision and Ophthalmology ARVO statement for the Use of Animals in Ophthalmic and Vision Research, and were approved by Case Western Reserve University IACUC (2011-0063). The protocol for the use of human peripheral blood from normal healthy volunteers was approved by the Institutional Review Board at University Hospitals of Cleveland (07-11-21). Informed consent was obtained in writing from each volunteer.

## Supporting Information

Figure S1 **Complementation of** ***A. fumigatus*** **and** ***F. oxysporum*** **mutant strains causes phenotypic reversion to WT parental strain phenotypes. A.** C57BL/6 mice were infected with 40,000 conidia from the WT *A. fumigatus* strain ATCC 46645 and the the complemented strains: sidA^R^, sidD^R^, sidF^R^, sidH^R^, sidI^R^, hapX^R^ and CFU analysis was performed at 48 h post-infection. **B.** C57BL/6 mice were infected with 30,000 conidia from the WT *F. oxysporum* strain 4287 and the complemented strain hapX^R^ and CFU analysis was performed at 48 h post-infection. **C.** *A. fumigatus* and **D.** *F. oxysporum* infected eyes were imaged at 24 h and 48 h post-infection.
(TIFF)

Figure S2 **Siderophores are required for survival of** ***Alternaria*** **during infection. A.** C57BL/6 mice were infected with 40,000 conidia from the *Alternaria brassicicola* strain Tf383, and the isogenic mutant strains: Δnps2 (no intracellular siderophores), Δnps6 (no extracellular siderophores), and Δnps2/6 (no intracellular or extracellular siderophores) and CFU analysis was performed at 48 h post-infection. **B.** Eyes were imaged at 24 h and 48 h post-infection **C.** Metamorph image analysis was used to quantify both cornea opacity area and **D.** total cornea opacity.
(TIFF)
