# Correction: Targeting Iron Acquisition Blocks Infection with the Fungal Pathogens *Aspergillus fumigatus* and *Fusarium oxysporum*

**DOI:** 10.1371/annotation/4f388450-90fe-4c65-9b9b-71a2b7935ac0

**Published:** 2013-07-23

**Authors:** Sixto M. Leal, Sanhita Roy, Chairut Vareechon, Steven deJesus Carrion, Heather Clark, Manuel S. Lopez-Berges, Antonio Di Pietro, Marcus Schrettl, Nicola Beckmann, Bernhard Redl, Hubertus Haas, Eric Pearlman

The seventh author's name was spelled incorrectly. The correct name is: Antonio Di Pietro.

