# Supplementary figures and images for: Targeting Iron Acquisition Blocks Infection with the Fungal Pathogens Aspergillus fumigatus and Fusarium oxysporum
Source: PLoS Pathog. 2013 Jul 11;9(7):e1003436. doi: 10.1371/journal.ppat.1003436 (PMC3708856; doi:10.1371/journal.ppat.1003436)

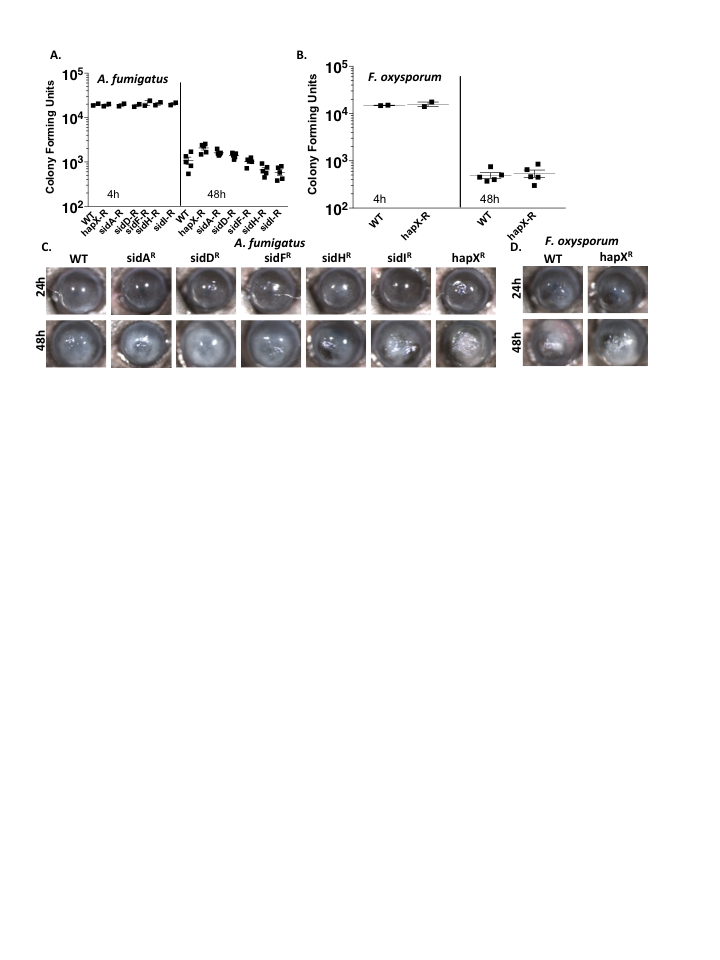

Supplement: Figure S1 — Complementation of A. fumigatus and F. oxysporum mutant strains causes phenotypic reversion to WT parental strain phenotypes. A. C57BL/6 mice were infected with 40,000 conidia from the WT A. fumigatus strain ATCC 46645 and the the complemented strains: sidAR, sidDR, sidFR, sidHR, sidIR, hapXR and CFU analysis was performed at 48 h post-infection. B. C57BL/6 mice were infected with 30,000 conidia from the WT F. oxysporum strain 4287 and the complemented strain hapXR and CFU analysis was performed at 48 h post-infection. C. A. fumigatus and D. F. oxysporum infected eyes were imaged at 24 h and 48 h post-infection. (TIFF) [file ppat.1003436.s001.tiff]

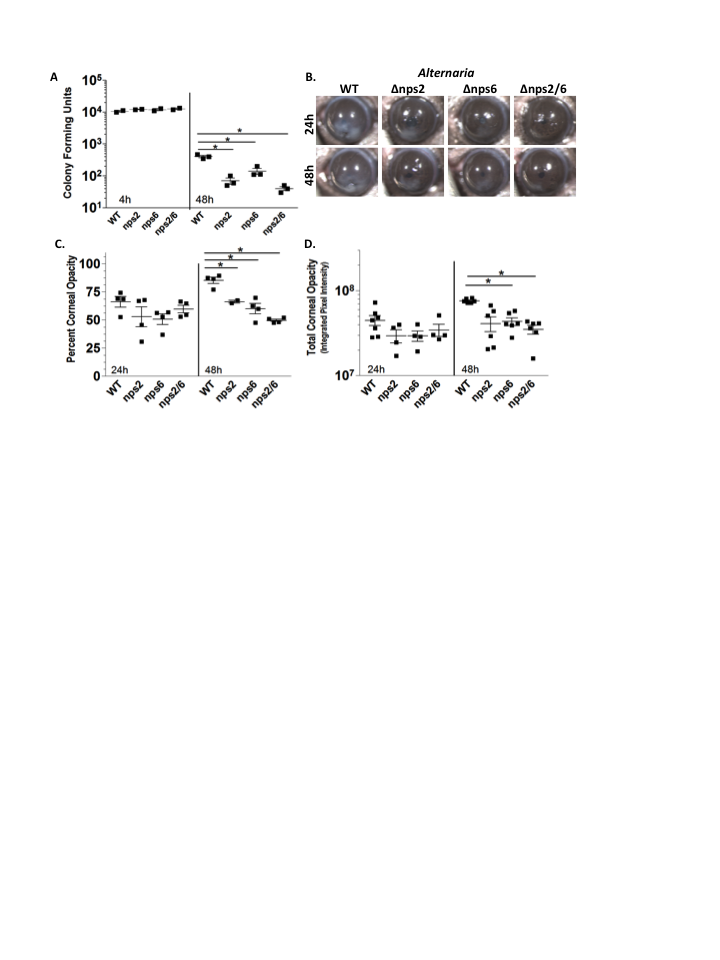

Supplement: Figure S2 — Siderophores are required for survival of Alternaria during infection. A. C57BL/6 mice were infected with 40,000 conidia from the Alternaria brassicicola strain Tf383, and the isogenic mutant strains: Δnps2 (no intracellular siderophores), Δnps6 (no extracellular siderophores), and Δnps2/6 (no intracellular or extracellular siderophores) and CFU analysis was performed at 48 h post-infection. B. Eyes were imaged at 24 h and 48 h post-infection C. Metamorph image analysis was used to quantify both cornea opacity area and D. total cornea opacity. (TIFF) [file ppat.1003436.s002.tiff]
